# Contract cheating: an increasing challenge for global academic community arising from COVID-19

**DOI:** 10.1186/s41039-021-00166-8

**Published:** 2021-07-30

**Authors:** Guzyal Hill, Jon Mason, Alex Dunn

**Affiliations:** 1 School of Humanities, Arts and Social Sciences, University of New England, Armidale, Australia; 2 International Graduate Centre of Education, Charles Darwin University, Darwin, Australia; 3 Charles Darwin University, Darwin, Australia

**Keywords:** Ghost-writing, Ghost-studying, COVID-19, Contract cheating, Online exams, Academic integrity, Global community

## Abstract

Due to COVID-19, universities with limited expertise with the digital environment had to rapidly transition to online teaching and assessment. This transition did not create a new problem but has offered more opportunities for contract cheating and diversified the types of such services. While universities and lecturers were adjusting to the new teaching styles and developing new assessment methods, opportunistic contract cheating providers have been offering $50 COVID-19 discounts and students securing the services of commercial online tutors to take their online exams or to take advantage of real-time assistance from ‘pros’ while sitting examinations. The article contributes to the discourse on contract cheating by reporting on an investigation of the scope and scale of the growing problems related to academic integrity exacerbated by an urgent transition to online assessments during the COVID-19 pandemic. The dark reality is the illegal services are developing at a faster pace than the systems required to curb them, as demonstrated by the results. The all-penetrating issues indicate systemic failures on a global scale that cannot be addressed by an individual academic or university acting alone. Multi-level solutions including academics, universities and the global community are essential. Future research must focus on developing a model of collaboration to address this problem on several levels, taking into account (1) individual academics, (2) universities, (3) countries and (4) international communities.

## Introduction

Plagiarism has existed for millennia, as has ghost-writing—one being a misdemeanour, the other a service (Dougherty, 2020). Over 2500 years ago, even the threat of the death penalty did not stop examinees cheating in the Chinese Keju examinations, with records revealing cheating behaviours such as the use of impersonators, bribery and elaborate cheat notes (Suen & Yu, 2006). Universities and academics worldwide have had to find solutions to plagiarism and are familiar with the existence of ghost-writing services for assessments. The problem has become so significant that the term ‘contract cheating’ has superseded ‘ghost-writing’ in the global university sector where the problem is increasingly recognised as a formidable challenge to the preservation of academic integrity (Lancaster, 2019; Walker et al. 2012).

Existing trends in consumer attitudes towards education, internationalisation and gaps in plagiarism software capabilities created an environment ripe for cheating when, due to COVID-19, universities with limited expertise with the digital environment had to rapidly transition to online teaching. This transition did not create a new problem but has offered more opportunities for contract cheating, diversified the types of such services offered and allowed for increased commercial contract cheating. While universities and lecturers were adjusting to the new teaching style, opportunistic contract cheating providers have been offering $50 COVID-19 discounts and students securing the services of commercial online tutors to take their online exams, or to take advantage of real-time assistance from ‘pros’ while sitting examinations.

The term *contract cheating* is used in this article to refer to a situation in which students can have their assignments and/or their exams commercially ghost-written due to an unprecedented move to online teaching formats; however, it is acknowledged that contract cheating may take many forms, and it does not always require money to be exchanged. It would be challenging, if not impossible, to identify how many students have used or are making use of these services, although Newton (2018) used 65 studies dating back to 1978 to identify a rise from a 3.52% historical average of cheating students to 15.7% post-2014, suggesting that such services may be having a significant effect on cheating behaviours.

Plagiarism software does not necessarily capture all occasions of plagiarism and may sometimes produce misleading results (Eaton et al., 2020; Walker, 2010). Forensic linguistic software offers opportunities to detect authorial anomalies; however, it is still being developed and cannot be regarded as infallible (Conlan et al., 2016; Daubner et al., 2020). Online examinations provide another avenue for contract cheating, and the literature suggests that students will cheat during online examinations if given the opportunity (King et al., 2009; Mata, 2020).

While invigilated in-person exams have historically been considered the only form of assessment wherein the identity of a student can be completely confirmed, the emerging service of online invigilated examinations has ameliorated these concerns somewhat, but such services are expensive and not without criticism, e.g., privacy concerns (Stewart, 2020; Zhou, 2020). As such, perhaps academics and universities should be less naive about academic integrity in COVID-19-affected teaching and beyond, particularly in light of empirical findings that academics frequently pass ghost-written assignments under the impression they were written by students (Lines, 2016).

Being less naive, however, comes at a cost and can place academics and universities in a precarious position. If the problem is discussed openly, evidence proving the fact an assignment has been ghost-written must be used, and action must be taken to penalise the students responsible. If such action is taken, it must be supported by the university, government and international community. Ensuring cooperation at such a large scale is challenging. Conversely, the price for not acting is higher and includes the erosion of academic integrity and the possibility that graduates will transfer their cheating tendencies to their workplaces. That is, the heightened development of contract cheating services and the rapid transfer to online examinations has broad implications for academic practice and institutional responses such as policy and procedures in universities internationally. To probe these issues more deeply, a literature review was conducted in which several key themes emerged. This was followed by a form of action research conducted in Australia, in which one of the authors assumed the role of a student seeking ways to cheat on assignment work.

## Literature review

### Trends: consumerism, internationalisation and gaps in plagiarism detection software

Education must be considered holistically in appreciating the contemporary context, which is often characterised by affordances of the digital environment; moreover, to ensure good governance and sustainability, the wider economic, social and technological dimensions surrounding academic integrity and misconduct must be taken into account (Goedegebuure & Davis, 2017). Prominent trends relevant for this research are consumer attitudes in higher education (economic), internationalisation (social) and the limitations of plagiarism-detecting software (technology). The trend of higher education accommodating a mass market (hence, consumer models) has been problematic, with higher consumer orientation being found to be associated with lower academic performance (Bunce et al., 2017). Universities are working on the bases of marketisation, competition, customer satisfaction and other consumer-oriented principles (Lesnik-Oberstein, 2015), and students are demanding customer ‘value’ from the universities (Woodall et al., 2014). In this way, education becomes viewed as a commodity (Gutting, 2015; Rawlings, 2015) rather than something earned through intellectual labour and through a process of improving skills in academic writing, critical thinking and communication.

Internationalisation also contributes to undesired consequences. International students ‘have less social, and particularly academic, support than their domestic peers’ (Grayson, 2008). Additionally, international students often lack study skills and English language fluency and experience cultural pressures to succeed, as well as the difficulties of living in a different culture for the first time (Simpson, 2016). This exposes them to stress and makes the option of ‘buying’ an assignment more attractive. Indeed, the issue with commercialisation of degrees in which students are, to some extent, also consumers (Bunce et al., 2017) is the fact that universities come to rely on income from international students, facilitating the development of unforeseen and unintended consequences. Universities must also be productive contributors to the economy, but significant improvements must be made before this can occur in an effective and efficient manner (Goedegebuure & Marshman, 2017). This is a dangerous situation wherein corruption is arguably becoming endemic, and long-held expectations of academic integrity are at serious risk of corrosion. Denisova-Schmidt (2017) highlighted that universities have an important function of socialisation—if students use commercial contract cheating services during their university study, they have the potential to transfer unethical or corrupt practices to workplaces and other areas of life where intellectual labour and skill are required and valued.

### Plagiarism and contract cheating

Plagiarism is usually defined within individual university academic misconduct policies. Generally, plagiarism occurs when the work of another is presented without acknowledgement. It often includes perhaps unintentional incorrect paraphrasing in more benign occurrences with intentional fraud in more malicious cases (Hexham, 2005). That is, it is ‘literary theft’ or ‘literary crime’ (Marsh, 2012). Self-plagiarism, or recycling of previously submitted work, is another area in which students might either intentionally or unintentionally breach misconduct rules. In legal terms, plagiarism may include fraud, forgery, false statements, conspiracy to defraud, proceeds of crime, breach of intellectual property laws and in some cases specific statutory offences (Feng, 1999; Linlin, 2011; Steel, 2017).

Universities use software systems such as Turnitin, SafeAssign, SNITCH, CrossCheck, AntiPlag and other related software to detect plagiarism, and these systems are constantly improving (Pàmieset al., 2020; Bolkan, 2006; Jiffriya et al., 2013; Niezgoda & Way, 2006; Walker, 2010; Zhang, 2010). However, currently, there is no fully reliable tool with which to detect all cases of plagiarism and contract cheating, and some researchers have developed systems to defeat plagiarism detection (Devore-McDonald & Berger, 2020). Existing systems have gaps that are being addressed but are not resolved at this stage (Bin-Habtoor & Zaher, 2012). Moreover, while promising developments have been made in forensic linguistics to ensure that academic integrity is enforced on a deeper level and breaches of academic misconduct are punished when required (Coulthard, Johnson, & Wright, 2016; Edwards et al., 2019; Peytcheva-Forsyth, Mellar, & Aleksieva, 2019; Sousa-Silva, 2020), technology is not being developed and implemented fast enough to curb all plagiarism practices, in particular, the bespoke commissioning of assessment tasks by non-students. As such, with or without the assistance of software, contract cheating remains a threat that must be considered by academics worldwide.

Ghost-writing practices are not new to academia. As early as 1960, ghost-writers were reproached for writing speeches (Bormann, 1961). In 2008, ghost-writing by researchers was litigated against (Ross et al., 2008). Academics and universities are aware of the existence of commercial contract cheating services for domestic and international students (Lines, 2016; Zheng & Cheng, 2015). The most notorious website, MyMaster, is renowned for causing an international scandal (McNeilage & Visentin, 2014; Visentin, 2015); some students who used it were expelled or suspended (McNeilage & Visentin, 2014). The website is no longer operational, but many other websites offer the same or similar services; many of which are owned by the same illegal enterprises but presented through different shopfronts (Ellis et al., 2018). Indeed, the business models of contract cheating individuals are becoming increasingly sophisticated, now extending from self-employed freelancers through to the sale of off-the-shelf web applications that incorporate all aspects of a contract cheating business (Ellis et al., 2018).

The term *contract cheating* is used in this article to refer to a situation in which students can have their assignments commercially ghost-written due to an unprecedented move to online teaching formats and the emergence of commercial ghost exam-taking services, though the term may also be applied to non-financial transactions (Lancaster & Clarke, 2008; Mahmood, 2009). The problem of contract cheating is often framed as a systemic failure as well as a corruption of student integrity standards (Chapman & Lindner, 2016; Daniel, 2016; Denisova-Schmidt, 2017; Kaktiņš, 2018). Further, Chapman considered the problem an international concern for governments, educators, students and other stakeholders (Chapman & Lindner, 2016).

While any form of plagiarism corrupts the academic system, contract cheating is particularly pernicious; in addition to breaching intellectual property laws and committing literary theft, the students involved undertake another, illegal form of organised commercial activity wherein someone assumes another’s identity, which amounts to committing fraud. Worryingly, the scale and sophistication of contract cheating practices are growing (Lancaster & Clarke, 2016). This is particularly concerning since students around the world admit to cheating with, for instance, 46% of students in Latvia admitting to purchasing a term paper (Danovskis, 2012).

### Impact of COVID-19 on assessments

On 11 March 2020, the World Health Organization Director-General declared COVID-19 a pandemic. A few weeks later, the Australian government introduced measures to limit its citizens’ exposure to the virus. All Australian citizens and permanent residents were prohibited from travelling outside Australia unless granted an exemption. Similar measures were implemented by countries across the globe. Universities in Australia and worldwide also had to appropriately manage the risks posed by the pandemic (Bao, 2020; Crawford et al., 2020; Khan & Jawaid, 2020; Wang et al., 2020). Most universities had no alternative but to transfer to online teaching, with the associated decision to conduct exams online. Notably, exams are currently the only form of assessment invigilated in person. Invigilation ensures that students’ identities are checked and that someone else is not taking an exam under a different ID. While there is some potential for corruption and fraud if twins or students who look alike take an exam under different IDs, the chance of this occurring on a mass scale is negligible.

One response to the COVID-19 pandemic has been for universities to employ commercial third-party online assessment services to invigilate, or proctor, examinations. These services may use humans, artificial intelligence and other digitally based tools to monitor students undergoing examination. One poll conducted in the wake of the pandemic suggested that 54% of the institutions polled used some level of online proctoring, with another 23% considering using such a service (Grajek, 2020). Based on reports, only 24 out of 47 Australian institutions used proctoring (Sankey, 2020). It must be recognised that, according to the above figures at least, a not inconsequential 46% of institutions are yet to use proctoring.

While these services provide many security measures unavailable to in-person invigilators, such as the use of biometric data and eye movement and keystroke tracking, they do so at significant cost to the institutions who use them—both financially and in terms of risks associated with breaching data protection and privacy legislation, not to mention the good will that might be lost with students who may feel the surveillance is so intrusive it breaches their basic rights (Grajek, 2020; Stewart, 2020; Zhou, 2020).

## Methods

To further investigate the findings from the literature, this study adopted a form of action research in which one of the authors assumed the role of a student and sought web-based services for cheating on assignment work. This made use of the online research method (ORM) and exploratory content analysis to identify the scope of contract cheating services available to students. In reporting on this, a secondary or consequent aim of the study is to alert lecturers and universities to the diversification and prominence of this dangerous practice.

The ORM operated at a basic level, using Google and Google Scholar as the primary search tools as they are widely available to students and lecturers (Granello & Wheaton, 2004). Advertised sites that typically appear in responses to queries were not considered because these depend on the budget of the organisations that advertise the services. Google, as a company, is using the ranking system calibrated by algorithms to bring most up-to-date and relevant results on the first page of the search page (Google, 2021).

Search queries were conducted for two generic terms ‘assignment help’ and ‘exam help’. The top five results were identified and analysed using exploratory content analysis methods in two points of time: mid-2020 (when pandemic was first declared and assignments or exams were due) and early 2021 (once the universities and students had time to adjust to new normal). The exploratory content analysis aspect allowed flexibility to include the necessary results (Elo & Kyngäs, 2008). The results of the websites were analysed using theme-based approach. The developed themes are presented in the ‘Results: COVID-19—from ghost-writing to ghost-studying’ section. These methods have been previously applied to research issues relating to ethics in management (Bell & Bryman, 2007) and academic dishonesty policy (Prenshaw et al., 2001); in this case, they were adapted to examine academic integrity and the ethics of student practices.

In probing this issue from a potential student perspective, one of the authors chose to go ‘undercover’ to discover firsthand the scale of available services and the processes involved in making use of them. ‘Undercover’ is used here in the sense of a lecturer posing as a student who needs ‘help’ with the assessment.

The limitation of this method is that the websites that can be found on Google are not the only providers of contract cheating services. As reported, contract cheating through a particular site-sharing website, Chegg, has increased by 196.25% during the pandemic (Lancaster & Cotarlan, 2021). This limitation, again, points to the scale of the problem because the providers of contract cheating services can be found offline, in file-sharing websites, through social media and websites offering gigs, such as Fiverr and essay mills—to name several other alternatives.

## Results

### COVID-19—from ghost-writing to ghost-studying

#### Commercial assignment ghost-writing: going undercover

The following text is an account of one of the co-authors:

As a lecturer, I used to have idealistic views of studying and education; it took several years to bring myself to investigate the services that are available to students. However, as unsavoury as the practice of buying assignments is, I believe that lecturers must at least be aware of it. So, in May 2020, after five years of teaching, I decided to go undercover to discover what options students can avail themselves of in terms of ordering assignments, and what barriers are in place in the same regard.

I faced an ethical dilemma in terms of whether to include in this article the names of the websites I found, since it would compile them in one location that students might find, and might serve as ‘advertising’ for unsavoury and, at times, illegal activity. However, the websites included in this article are the top responses to a basic Google search and students willing to order ghost-writing services will have no difficulty finding them online. Further, contract cheating businesses often use aggressive marketing tactics to reach students (Bretag, 2017). Lecturers and universities, however, are less likely to search for these services and might be unaware of this academic black market that is also available through social media.

A Google search for the term ‘assignment help’ returned 279,000,000 results in 2020 and 302,000,000 in 2021. The following commercial contract cheating websites appear on the first page in the following order (based on a search conducted in mid-June 2020 and early 2021 from Australia):

1. https://myassignmenthelp.com/. My Assignment Help is rated 4.9/5, based on 14,001 reviews, and has an instant chat function. The sample assignments are grouped by university (e.g. Charles Sturt, Victoria University). A $20 instant cashback offer was made, and a quote for services depended on the word count and deadline. There is a loyalty programme, a chance to win $2000 and a $2 reward for providing feedback. My user number was 419,705, so presuming this represents the number of users it suggests almost half a million students worldwide have used or are using the service. The website was established in 2007. It ranks as the first website Google search results in both 2020 and 2021.
2. https://assignmenthelp4me.com/. Assignmenthelp4me is described as delivering high-quality assignment solutions, facilitated by their ‘substantial work ethics’ and supported by their global team of 5000 writers and 26,000 academic assignment helpers. Assignmenthelp4me claims to help students in Australia, Canada, India, New Zealand, the United Kingdom (UK) and the US. This ranks as the second website in Google search results 2020 and in 2021.
3. https://www.myassignmentservices.com/. My Assignment Services, at the time of searching, offered a $50 COVID-19 discount and a 15% discount during World Environment Week, with an accompanying ‘save the planet’ slogan. Furthermore, if students order in bulk, they receive a 50% discount. The website boasts the following: ‘Quality Work: We never compromise on quality. Affordable Pricing: We understand the stringent budget of a student. Plagiarism-Free: Plagiarism is a big NO when it comes to academic writing. Time-Saving: Submitting assignments after the submission time can attract a penalty.’ There is also a high distinction grade guarantee, or the client will reportedly receive their money back. The website claims to have been operating since 2010. My Assignment Services lists prominent Australian universities and includes information regarding assignment writing for particular universities. For example, about Monash University, the website states: ‘if you are seeking the best assignment writing services in Monash at reasonable prices, there is no better place than My Assignment Services. Just log on … to get in touch with us now’. According to a quote provided on the website, a 2000-word essay for an Introduction to Business Law unit costs AU$154.08 or AU$138.67 with the COVID-19 discount, due to service charges. An additional discount was received when we did not engage through email, further lowering the price to AU$130.97. Notably, the misleading advertising regarding the $50 discount did not translate into reality, highlighting the vulnerabilities that students are exposing themselves to by using these services. This ranks as the third website in Google search results in 2020 and 2021.
  - In 2021 https://www.instantassignmenthelp.com.au/ comes third on the page with 50% COVID-19 discount still offered. The ‘speedy delivery’ and 24/7 support is emphasised. Logos of major universities are displayed on the first page to add credibility, and plagiarism report is offered for additional fee.
4. https://www.thanksforthehelp.com/. Thanks for the Help offers $100 instant credit. The assignments written through this service have a no-plagiarism guarantee: ‘every assignment … under our Assignment Help service goes through Turnitin that ensures that no assignment is plagiarised.’ In addition, the service is diversifying to online exams: ‘ever stuck in a situation where an assignment is due in 6 hours and there is no one to help you? If yes, then use [our services].’ The services are offered for the following countries: the US, Australia, the UK, the United Arab Emirates, Canada, Malaysia, Singapore and Ireland. This ranks as the fourth result on Google search results in 2020 and fifth in 2021.
5. https://www.allassignmenthelp.com/. AllAssignmentHelp claims to be the world’s number one assignment help service. In answer to the frequently asked question of whether professors will discover students’ use of the service, the website claims: ‘as long as you stick to our fair use policy, you should not worry about anything. We provide 100% unique assignment solutions; hence, they will not flag in your college.’ The site has a 4.92/5 rating, and nine out of 10 students claim that AllAssignmentHelp assists them to achieve better grades. Testimonials on the website include, ‘I was panicking about my Medical homework that was due … Eventually, I came across allassignmenthelp.com … Within a day I got my work. Moreover, I received a cashback from them. They are truly the saviours, kudos to the experts’ and ‘so far I have found allassignmenthelp.com are very professional. Their customer support is active and available 24/7. Within a day I got my Civil engineering assignment. I keep coming back to them only due to this quality.’ This ranks sixth on Google search results in 2020 and fourth in 2021.

We followed up and examined the first five results in detail. All websites were easy to use, convincing with state-of-the-art marketing and website design tools, with the exception of Assignmenthelp4me. The websites had mobile contact numbers, WhatsApp assistance and persistent online chat options. Before undertaking this research, we were under the impression that assignment ghost-writing was not so widespread and such a mainstream service; however, we now understand that such services are readily available. The websites also used referrals, discounts, gamification, artificial intelligence and other functions that universities, in some cases, are only beginning to introduce. Further, we used to think it would be difficult for students to use these services. However, to the contrary, the service providers removed all possible friction points. They were approachable, user-friendly, included discounts and testimonials and were eager to disburse any doubts regarding the legitimacy and quality of their services.

In addition to the more ‘professional’ contract cheating services that link numerous clients with numerous ghost-writers, individual ghost-writers and their clients also use freelance marketplace platforms such as Fiverr.com to engage in one-to-one contract cheating transactions. In a study of this platform in regard to ghost-writing services, it was found that ‘96 providers have generated around US$270,000 of essay writing business between them’ (Lancaster, 2019). Now, more than ever, it is easier to link customers with service providers on a global basis and the contract cheating business is no exception as it includes numerous providers and marketplaces for those seeking to cheat. Perhaps universities and academics should be less naive about integrity and academic standards in COVID-19-era teaching and beyond?

### Commercial exam ghost-studying

IIn terms of online examinations, a review of the literature led Brothen and Peterson (2012) to formulate the following hypothesis: given the opportunity to gain points, students will cheat during examinations. In a US study, 73.6% of business students reported that cheating during online exams is easier (King et al., 2009). Another US study found that university staff suspected online cheating, but the majority did not proactively implement relevant prevention measures (Rogers, 2006). From a more optimistic perspective regarding the introduction of online exams, some research found evidence ‘that the difference in the testing environment creates a disadvantage to students taking the online exam which somewhat offsets the advantage that the unproctored students gain from greater opportunities to cheat’ (Fask et al., 2014).

A Google search for the terms ‘online exam help’ returned 538,000,000 results in 2020 and 559,000,000 in 2021. The following commercial ghost-studying websites appear on the first page in the following order (based on a search conducted in mid-2020 and early 2021 from Australia):

2020

1. https://www.onlineassignmentexpert.com/best-online-quiz-help.htm/. At the time of searching, Online Assignment Expert offered a discount of up to 50% for services due to COVID-19. The site offers services for nursing, economics, law, statistics and other online exams. It boasts confidentiality, 24/7 support and a no-plagiarism guarantee.
2. https://www.brainyassignmenthelp.com/online-exam-help/. Brainy Assignment Help has the slogan ‘pay someone to take my exam’. The website sells their services as being understanding of the pressures that students face: ‘students across the globe favour Online Exam Help because it is the easiest medium for obtaining concept clarity. Parents and friends may be unable to help you … and the fees charged by a private tutor can be a real financial burden.’ In this way, the services provided are portrayed as students’ only option. The units covered include accounting, economics, biology, humanities, psychology, mathematics, statistics, business management, chemistry, physics and sociology.
3. https://www.myassignmentservices.com/. My Assignment Services also provides both exam services. The following is a list of online quizzes for which help is available: nursing online quizzes, human resources management online exams, marketing weekly quizzes, public health quizzes, accounting online exams, engineering online exams, business law quizzes, MYOB/Perdisco online tests, management quizzes, bioinformatics online exams, genetics quizzes, epidemiology online quizzes, physics online exams, biology online exams and chemistry quizzes.
4. https://assignmentgeek.com/online-test-help.html. Assignmentgeek is similar to the above services and has the slogan of ‘the fastest way to better grades’. The website advertises the services by stating: ‘You don’t have to waste time trying to solve problems yourself…. Your information is safe and secure with our team focused on providing the tools and custom content necessary so (sic) you can succeed.’ The assistance is available 24/7 ‘assistance is available within minutes; you can work with a trusted helper that assists each step of the way during the writing process’.
5. https://takemyclassesonline.com/take-my-exam-help.php. Take My Classes Online runs under the slogan ‘we have the best experts to do online exams for you. Ask us to take my exam for me!’ and claims that PhD-qualified tutors will assist students. The prices are US$25 for 1000-word assignments, US$79 to complete a submission for a class and US$599 to complete a course.

Unlike assignment help results, the online exam help exam results changed in 2021, with the following websites appearing on the first page:

1. https://www.myassignmenthelp.net/online-exam-help My assignment help claim to be is the best assignment service provider offered online by a team of expert tutors of Australia, the UK and the USA’. The site also claims that they have checked 324,987+ assignments, have 5481+ PhD experts and offer services for 157+ subjects.
2. https://www.thetutorshelp.com/online-exam-help.php runs under the slogan ‘Online Exam Help - Pay Someone to Do My Online Exam’. The website offers payment plans and 24/7 h services.
3. https://myassignmenthelp.com/online-exam/ is the same services that appears for assignment help and was not active in searches for exams in 2020. In 2021, the website appears in searches for both categories.
4. https://www.onlineassignmentexpert.com/best-online-quiz-help.htm This website used to be number 1 in 2020.
5. https://www.myassignmentservices.com/online-quiz-help.html This website used to be number 3 in 2020.

It is difficult to gauge the number of students who have used or will use such online exam help services, but it can be observed that both searches returned increased number of results. The increase in assignments market was by 23,000,000 and exam help market by 21,000,000. It is possible that the websites use misleading advertising to create the impression that the services are used frequently, despite low actual use rates. Indeed, ‘slick advertising is not necessarily borne out in reality’, with the potential for misleading practices, flawed promises and service delivery failure (Sutherland-Smith & Dullaghan, 2019). This is an area of illegal activity that must be watched closely by the academic community. The implications of not tracking this academic black market are examined below.

## Discussion and implications for the global academic community

The results demonstrate nimble and adaptable response of illegal contract cheating websites to the global pandemic. In addition to nimbleness and adaptability, the websites demonstrated longevity with almost the same Google results appearing for assignments. What is required, in these dire circumstances, is systematic organised action to address a systemic issue that is growing rapidly and persisting on a global scale. The blame cannot be simply placed on individual lecturers, institutions or even countries. Therefore, the discussion section begins by identifying the victims of this race to the bottom and progresses to identify some solutions on several levels, taking into account academics, universities and global community.

**Fig. 1:**
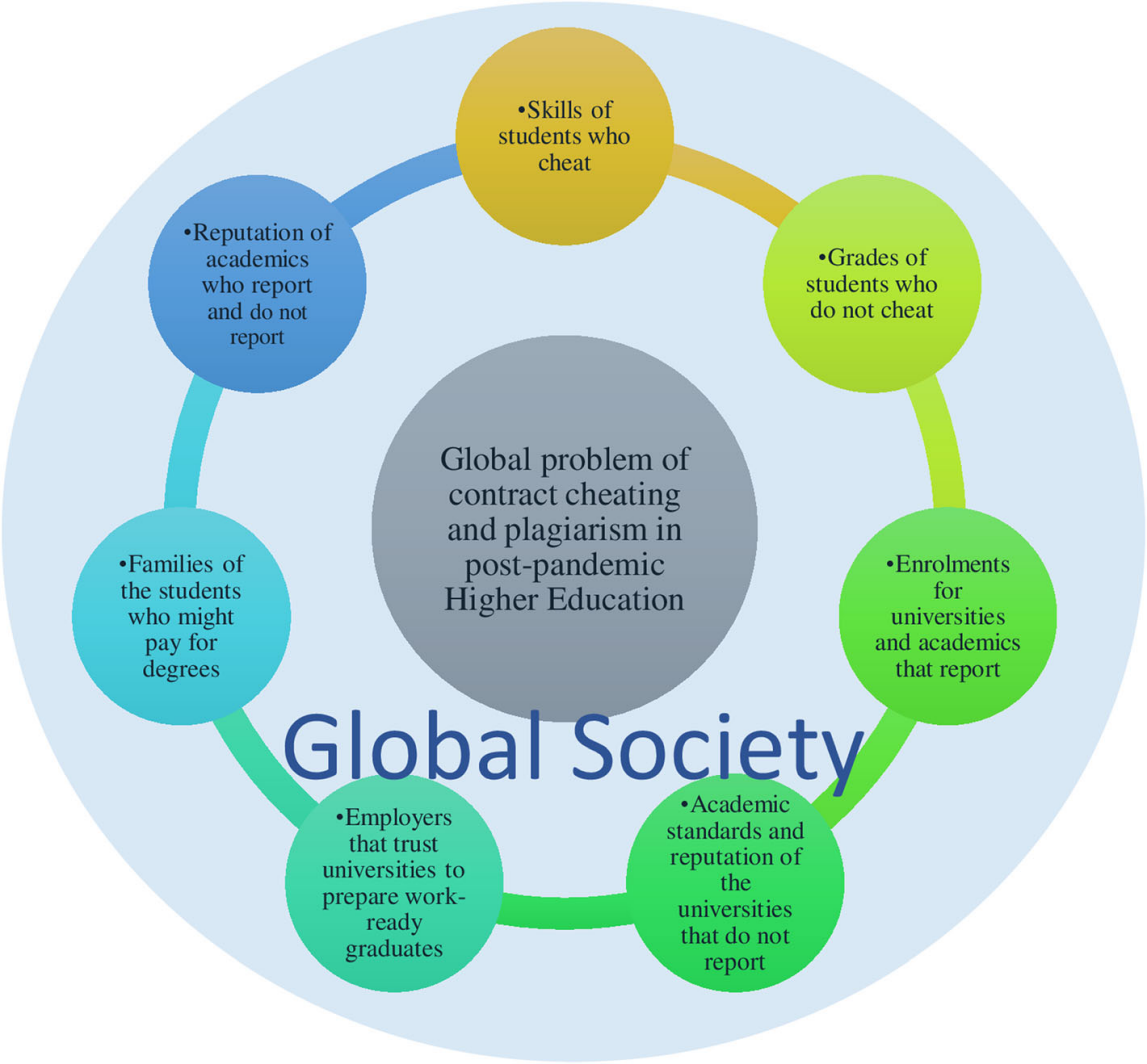
Growing scope of impact of contract cheating in higher education

There are no winners of this race to the bottom when it comes to the global problem of contract cheating and plagiarism in post-pandemic higher education. Figure 1 depicts the growing scope of impact of contract cheating. The students who use the ‘services’ do not learn valuable skills, the students who do not use the services observe the cheating students receiving grades without necessary effort. Students do not learn new skills and have even been blackmailed when trying to enforce the ‘high marks guarantee’ advertised by illegal websites (Lancaster, 2016) which becomes an equity issue for the university. The academics who detect and report the conduct are losing valuable resources that can be allocated to development of teaching materials, academics who do not detect and report are seen as naive by students. Universities that choose to investigate and prosecute the breaches might have lower enrolments and be seen as ‘too tough’ while universities that choose not to investigate might face the problem of lowering academic standards. In the long run, the employers cannot trust universities for preparing skilful work-ready graduates. The global society at large suffers from ignoring the growing scale of the issue because academic integrity is corrupted, with the potential for students, providers, industry and the international community to lose trust in education standards.

Due to the scale of the problem, multi-level solutions are required including academics, universities and global community.

### Academics

Academics are at the frontline of preventing, detecting and often investigating contract cheating. An Australian study found that ghost-written papers were generally passed by academics, who thought they were grading real students’ work (Lines, 2016). Conversely, Dawson and Sutherland-Smith (2018) found that, when alerted to the potential presence of ghost-written assignments, markers ‘detected contract cheating 62% of the time’ and real student papers 96% of the time. In another study, when lecturers were alerted to contract cheating and trained to detect it, the detection accuracy increased from 58 to 82% (Dawson & Sutherland-Smith, 2019). Therefore, knowledge of these practices on behalf of academics worldwide is so important. One of the biggest problems is not detection, though, it is the difficulty of proving a breach, the administrative burden of investigations, and a lack of confidence that appeals will be denied, all leading to a reluctance to report in the first place. One could imagine this is exacerbated by large class sizes (getting to know your students being one of the best ways to detect breaches) and a casualisation of the workforce.

The authors note that the pandemic may have led to some positive long-term developments in relation to academic integrity as well as exacerbating the immediate challenges that are the focus of this article. Indeed, a collection of reflections from the *Canadian Perspectives on Academic Integrity* journal suggests that the crisis has forced more academic and learning and teaching staff to consider academic integrity when designing online courses and assessments (Bens, 2020; Nearing, 2020; Scurr, 2020). The link between assessment design and contract cheating in terms of both opportunities to cheat and motivations associated with dissatisfaction with the learning and teaching environment has been clearly established (Bretag et al., 2019), and there is no doubt many teaching professionals have been addressing that link both pre- and post-pandemic. As such, good course and assessment design that is appropriate to the medium in which it is delivered is undoubtedly one part of the solution to the problem of academic misconduct and remote learning.

This paper is about raising awareness and as such a comprehensive guide to preventing, detecting and investigating academic misconduct in relation to contract cheating, if it were possible, is outside its scope. However, there is a wealth of resources available. For example, the Australian Tertiary Education Quality Standards Agency has produced the evidence backed *Substantiating contract cheating: A guide for investigators* (Curtis, et al., n.d.), which provides a range of resources for investigators such as guides of interviews as well as a list of signals that might indicate misconduct. Examples of such signals include very high or very low scores on text matching software, checking document properties, content not appropriate to discipline area, quality alters from expectations, language use and ability, unreadable language such as jargon or misused words, reference list but no in-text citations, irrelevant sources, falsified references, work that does not meet the set criteria, reflections are done badly, check IP addresses of submissions and check student analytics for little or no engagement other than submission (Curtis, et al., n.d.). As this list suggests, training is essential for academic and professional staff who deal directly with academic misconduct. Furthermore, encouraging staff to stay in positions relating to academic integrity means that those staff will build expertise that can then be shared with their peers.

### Universities

Awareness and detection of contract cheating by academics alone is not enough to ensure that universities effectively deal with the problem and systemisation through sound policies and processes that are clearly communicated, as well as their consistent application within often diverse institutions, are also fundamental to maintaining academic integrity within and across universities. The dynamics involved in both contract cheating and university cultures are complex and nowhere is that complexity more obvious than in devising university policy and processes that not only react to misconduct, but also seek to proactively address it. Policy can dictate everything from the type of technology employed to combat cheating and the training provided, or not, through to the design of courses and assessments. Good policy should create a culture and systems of integrity that is clear, systematised, adequately resourced and just. Though the details of good policy are too numerous to recount here, a range of tools to assist with such a task has been formulated, with the Exemplary Academic Integrity Toolkit being one example (https://lo.unisa.edu.au/course/view.php?id=6751). Universities may also choose to emphasise and embed a values-based approach such as the one advocated by the International Center for Academic Integrity through its six fundamental values of honesty, trust, fairness, responsibility and courage (ICAI, , n.d.). An institution’s public commitment to such principles raises and maintains awareness amongst staff and students about academic integrity and misconduct.

Of course, with more benign forms of plagiarism, there is room for argument that students should be educated about the skills of paraphrasing and referencing, rather than penalised (Blum, 2009). The advantages of embedding academic integrity material in courses and careful assessment design can also be encouraged or mandated by universities (East, 2016). For example, without claiming that cheating can ever be designed out, a survey of eight Australian universities found that students are prone to rationalising cheating when there is a perceived lack of care or interest from academic staff or the university, and as such, it recommends building relationships with students as it builds trust but also helps in detection as staff reported that they detected over 70% of cases due to knowledge of the student (Harper et al., 2019). This suggests that the course and assessment design should not only strive to meet learning objectives in an engaging way, but it should also foster direct interaction between student and teacher—something that both students and teachers are finding a challenge in the post-COVID-19 transition to distance learning. The study also found that while cheating cannot be designed out of any course, the appropriate use of authentic assessment can make cheating harder and easier to detect. Assessment by examination remains an important part of university assessment practice, and as such, it too needs to adapt to an online environment in a way that ensures, or even increases, integrity.

Notably, online invigilation is subject to the resource availability of an individual university. The available literature on COVID-19-related assessment changes suggests an increase in online invigilation, which may or may not be cost-effective for universities as institutions (Khan & Jawaid, 2020; Nizam et al., 2020). Apart from the significant cost of purchasing an externally provided invigilation service, institutions have found that successfully scaling up invigilated online examinations requires the allocation of significant resources in terms of training their own staff and students to use, and have confidence in, such systems (Day & Lawrence, 2020). Online invigilation may well offset many concerns surrounding identification verification through using multi-factor identity checks, keystroke analysis and artificial intelligence to track suspicious facial movement, lighting changes, etc. (Proctoru, , n.d.); however, such measures have attracted negative media attention relating to both potential privacy breaches and the potential commodification of student information (Sky News Australia, 2020; Zhou, 2020). In other words, online invigilation providers are not criticised for being too lax on identification confirmation, but for being too intrusive. As such, universities should recognise that COVID-19 has accelerated the need for careful consideration of the way technology, academic integrity and assessment practices intersect on a larger scale than has previously been considered.

While online invigilation services provide institutions a certain level of security against exam misconduct, many institutions have not had the means nor the time to implement them appropriately at the scale required by the urgent demands of the COVID-19 pandemic (Hillier, 2020), and as such, they use inappropriate technology and practices to assure academic integrity. The primary concern for such institutions is that no lecturer can be confident that the person taking an exam is the intended student and not someone else with the student’s login. No lecturer can ensure that only one person is taking the exam and that there are not multiple students in the same room. This short-term solution to a pandemic situation might have far-reaching implications for the integrity of the education system worldwide. A less conventional but more viable option conducive to preserving academic integrity would have been hiring large centres to ensure social distancing or conducting exams outdoors with an increased number of invigilators. Even if online exams are required, these could be conducted in a computer lab setting with invigilators present and social distancing measures implemented. A further issue with uninvigilated online exams is that students cannot be prohibited from using their mobile devices, which can induce misalignment and distraction (Barry et al., 2015) and facilitate communication with third parties.

### Global community

The literature on plagiarism is unequivocal—plagiarism is ‘a global issue’ (Butakov & Scherbinin, 2009; Cameron et al., 2012; Roberts, 2007; Sutherland-Smith, 2008). Plagiaristic practices and systems that transcend state borders are also causes of alarm for international organisations. The UNESCO International Institute for Educational Planning and the International Quality Group of the US Council for Higher Education Accreditation have created the Advisory Statement for Effective International Practice to alert and inform governments (Daniel, 2016). The Advisory Statement suggests measures for preventing and combatting corruption in admission, assessment and award practices worldwide. In addition to publishing and enforcing the codes of conducts, the Advisory Statement recommends encouraging whistle-blowing practices, a measure that can be encouraged by universities. Whistle-blowing must be encouraged by both academics and students. To achieve this, it might be necessary to conduct anonymous surveys after exams to discover whether students observed other students partaking in contract cheating practices. The universities can place an anonymous form online to allow whistle-blowers report academic dishonesty.

The unique circumstances induced by technology and COVID-19 measures heighten the threat to integrity, for example, the rapid shift to online examinations leading to assessment practices unsuitable for online environments being employed and a consequent jump in test scores (Eaton, 2020). This results in a disconnect between personal skill and grade and an environment of distrust between students and university staff. (Kaktiņš, 2018) highlighted the danger of future employers distrusting university qualifications if the practice of contract cheating continues.

The commercial scale of contract cheating services offered online presents a threat to the global academic community. While international organisations, universities and the academic community are adjusting to new, COVID-19-affected, teaching practices, these issues must be brought to the attention of individual academics so that the problem can be addressed at the grassroots level. On a more pessimistic note, the illegal services are developing at a faster pace than the systems required to curb them: ‘detecting contract cheating is becoming more difficult, as students and intermediary contractors become more sophisticated’ (Clarke & Lancaster, 2013). Moreover, it seems that illegal websites have significantly benefited from COVID-19’s disruption to tertiary education.

The perceived threat of contract cheating is so serious that governments are increasingly legislating against the practice, though the challenges of making such laws effective are considerable. Amigud and Dawson (2019) report that while a number of jurisdictions including a number of US states, Ireland, New Zealand and most recently Australia have legislated against contract cheating, the efficacy in such laws reducing contract cheating is questionable. After surveying 17 US states that had legislated against contract cheating, the authors found that prohibition had no noticeable effect on cheating behaviours, leading them to conclude that the focus of preventative efforts should not be on the supply side of contract cheating, but rather on universities, academics and students (Amigud & Dawson, 2019). As Draper and Newton (2017) suggest, the common perception of contract cheating is that it only takes place between three parties, the institution, the university and the person hired, but in actuality, it may be many more ‘a company regulated by a government, hosted on a website, with advertisers and advertising a bidding system with multiple writers etc.’ (p. 7). Legal remedies to the problem might be further complicated when one considers that each of these actors could be in different countries (Draper & Newton, 2017). Nevertheless, Draper and Newton (2017) suggest that pursing new laws designed to combat cheating are ethically desirable, practically achievable and in the public interest. While jurisdictional and other enforcement issues will always exist with any legal approach to contract cheating, the increasing rate of legislation at the very least sends a clear message to students and institutions across the world about the seriousness of contract cheating.

## Conclusion

The issues addressed in this research concern ethics, academic integrity and illegal commercial activity. There is a certain spectrum of plagiaristic activity, with the darker side of contract cheating involving criminal activities of assuming and allowing the assumption of another person’s identity for the purposes of committing fraud. This problem is not limited to a particular university or country; it is international, and as such, it poses considerable jurisdictional issues in enforcing laws. Further, the possibility of committing crime in this regard has been exacerbated by the transfer to online teaching across the whole education sector as a result of COVID-19.

The existing problems of consumer attitudes to education, internationalisation and gaps in plagiarism detection are the drivers of diversification of contract cheating services. These services undermine the value of education and academic integrity standards and, more importantly, undermining the role of education in preparing professionals for a workplace. This leads to a problem of global proportions that must be collaboratively addressed by universities and academics, both while technology is being developed to curb these practices and after such development, with a constant need to remain alert to new developments that could threaten academic integrity. The global scale of the pandemic has accelerated the digital disruption of traditional teaching and assessment practices across international boundaries, but in so doing, it has also created an environment where most providers are unprepared for such a rapid shift into large-scale online education delivery. As a consequence, some individual institutions are ill-equipped to design, deliver and monitor assessments to deter and avoid academic integrity breaches in the face of the sophisticated and easily accessible businesses ready to take advantage of such a disruption.

In raising awareness about the issues emerging from current practices in contract cheating, the scale of the problem suggests it cannot be addressed by an individual academic acting alone and clarifies some directions for multi-level solutions. Future research must focus on developing a model of collaboration to address this problem on several levels, taking into account (1) individual academics, (2) universities, (3) countries and (4) international communities. What is required is the proposal of a systematic organised action to address a systemic issue on a global scale.

## Data Availability

Not applicable.

## Abbreviations

COVID-19: Coronavirus disease of 2019
ID: Identity document
US: United States of America
UK: United Kingdom
UNESCO: United Nations Educational, Scientific and Cultural Organisation
