# Correction to: Contract cheating: an increasing challenge for global academic community arising from COVID-19

**DOI:** 10.1186/s41039-021-00173-9

**Published:** 2021-08-27

**Authors:** Guzyal Hill, Jon Mason, Alex Dunn

**Affiliations:** 1 Asia Pacific College of Business and Law, Charles Darwin University, Darwin, Australia; 2 College of Indigenous Futures, Education & Arts, Charles Darwin University, Darwin, Australia; 3 School of Humanities, Arts and Social Sciences, University of New England, Armidale, Australia

## Correction to: Research and Practice in Technology Enhanced Learning (2021) 16:24 10.1186/s41039-021-00166-8

In the publication of the original article (Hill et al. 2021), the authors’ affiliations were found to be incorrect.

The correct affiliations of the authors are:

1. Asia Pacific College of Business and Law, Charles Darwin University, Australia
2. College of Indigenous Futures, Education & Arts, Charles Darwin University, Australia
3. School of Humanities, Arts and Social Sciences, University of New England, Armidale, Australia

The original paper has been updated.
